# Health-related fitness benefits following concurrent high-intensity interval training and resistance training in patients with type-1 diabetes or type-2 diabetes

**DOI:** 10.3389/fphys.2024.1466148

**Published:** 2024-11-20

**Authors:** Corentin Scoubeau, Malgorzata Klass, Bert Celie, Chantal Godefroid, Miriam Cnop, Vitalie Faoro

**Affiliations:** ^1^ Cardio-Pulmonary Exercise Laboratory, Faculty of Motor Sciences, Université Libre de Bruxelles, Brussels, Belgium; ^2^ Research Unit in Biometry and Exercise Nutrition, Faculty of Motor Sciences, Université Libre de Bruxelles, Brussels, Belgium; ^3^ Laboratory of Applied Biology and Research Unit in Applied Neurophysiology, Faculty of Motor Sciences, ULB Neuroscience Institute, Université Libre de Bruxelles, Brussels, Belgium; ^4^ Department of Cardiology, Erasmus Hospital, Brussels, Belgium; ^5^ ULB Center for Diabetes Research, Faculty of Medicine, Université Libre de Bruxelles, Brussels, Belgium; ^6^ Division of Endocrinology, Erasmus Hospital, Brussels, Belgium

**Keywords:** aerobic capacity, body composition, high intensity interval training, VO_2_peak, glycated hemoglobin, trainability, diabetes mellitus, VO_2_max

## Abstract

**Introduction:**

Cardiorespiratory fitness (CRF), as assessed by VO_2_peak, along with metabolic and cardiovascular health indices, represents the strongest predictors of survival. However, it remains unclear whether concurrent high-intensity interval training (HIIT) and resistance training (RT) can similarly enhance these health markers in patients with type-1 diabetes (T1D) or type-2 diabetes (T2D) compared to healthy individuals.

**Methods:**

Adults with uncomplicated T1D or T2D and healthy normoglycemic controls matched for sex and age (HC1 and HC2) performed 3 training sessions/week of concurrent HIIT and RT for 12 weeks. Pre- and post-intervention assessments included: lipids and glycemic profile, body composition (dual-energy x-ray absorptiometry) and a cyclo-ergometric cardio-pulmonary exercise test.

**Results:**

Training improved VO_2_peak, the ventilatory threshold (VT1), maximal workload, ventilation and O_2_pulse, similarly in T1D in HC1 without changes in body composition or glycemic profile. In patients with T2D, training improved insulin sensitivity (HOMA-IR), lean mass, VE/VCO2 slope, VT1 and maximal O_2_pulse, workload and VO_2_peak with reduction in fat mass and visceral adipose tissue (VAT) (all, *p* < 0.05). However, improvements in VO_2_peak and O_2_pulse were lower than in healthy controls (respectively, T2D: +9%, HC2: +18% and T2D: +6%, HC2: +19%, *p* < 0.05).

**Conclusions:**

Both patients with T1D and T2D benefit from combined HIIT and RT by improving CRF with specific adaptations influenced by the presence and type of diabetes. While identical magnitude of achievements were observed in T1D and HC1, T2D patients exhibited lower VO_2_peak and maximal O_2_pulse improvements but associated with notable additional health benefits regarding insulin sensitivity, body composition, visceral adipose tissue and ventilatory efficiency.

## 1 Introduction

Diabetes mellitus currently affects 537 million people, with an estimated worldwide prevalence of 10%, and an expected increase up to 783 million people by 2045 (International Diabetes Federation, 2022). The disease is associated with high morbidity, absorbs 12% of the total healthcare expenditure in developed countries and caused 6.7 million deaths in 2021 (International Diabetes Federation, 2022). Diabetes is defined by a chronic elevation in blood glucose, with heterogeneous etiology (type 1, type 2, monogenic forms) and progressively leads to common structural and functional complications (Cole and Florez, 2020). Most frequent complications include both microvascular (e.g., retinopathy, nephropathy, neuropathy) and macrovascular diseases (e.g., premature atherosclerosis, coronary artery disease), all of which contribute significantly to increased morbidity and mortality (Maranta et al., 2021).

A large body of evidence shows that physical exercise is a safe and effective strategy to counteract cardio-vascular complications in patients with diabetes (Grace et al., 2017). Exercise training induces multiple systemic effects by evoking physiological adaptations that act specifically on diabetes management targets. For instance, enhanced insulin sensitivity and glucose uptake by skeletal myocytes have been observed in exercising individuals with diabetes which clearly improved glycemic control in this cohort (Støa et al., 2017). Our research group among others recently highlighted a direct effect of exercise training on pancreatic beta cell protection from apoptosis through circulating mediators in both healthy individuals and patients with diabetes (Coomans de Brachène et al., 2023). These findings suggest a protective role for exercise at the beta-cellular level, which could contribute to long-term glycaemic control and the prevention of complications. In addition to glycemic benefits, exercise training significantly improves both microvascular and macrovascular function by enhancing endothelial function, reducing atherosclerosis, lowering inflammation, improving vascular remodeling as well as mitochondrial functioning and biogenesis, reducing oxidative stress, and enhancing blood flow. Due to the above mentioned muscular and cardiovascular adaptations, an improved glycaemic control and/or insulin resistance with reduced cardiovascular risk factors [lipid metabolism, body composition, blood pressure, and aerobic capacity (VO_2_peak)] will take place (Cosentino et al., 2020). Notably, a low VO_2_peak is an independent risk factor of cardiovascular disease and all-cause mortality in the general population, and patients with diabetes in particular (Kurl et al., 2022).Thus, implementing interventions to improve aerobic capacity and body composition are therefore essential to reduce cardiovascular risk and delay onset complications (Balducci et al., 2012).

Despite the well-established scientific and clinical evidence supporting the effectiveness of exercise interventions, patients with T1D and T2D generally display an impaired VO_2_peak compared to healthy participants. Several pathophysiological mechanisms contribute to this abnormality, including cardiogenic, vasculogenic, mitochondrial and neurogenic factors, which may act independently or in an integrated manner (Wahl et al., 2018). At the cardiac level, impaired diastolic function and vascular stiffness limit maximal stroke volume by reducing pre-load and increasing afterload (Rissanen et al., 2015). Autonomic neuropathy, common in both T1D and T2D, can impair chronotropic regulation at exercise, with some evidence suggesting that chronic hyperglycemia may desensitize beta-adrenergic receptors thereby reducing maximal heart rate (Turinese et al., 2017; Moser et al., 2018). Moreover, heterogeneity in muscle oxidative function as well as perfusion and the increased O_2_ affinity of HbA1c for O_2_ will reduce peripheral oxygen extraction in O_2_ and further limit aerobic capacity in both T1D and T2D (Ditzel, 1976; Nesti et al., 2020). Importantly, previous observations, although limited by the scarcity of studies, also indicate that both patients with T1D and T2D may experience a blunted ability to train aerobic capacity (Minnock et al., 2022; Burns et al., 2007). However, this lower training induced response was only observed in T1D patients with baseline mitochondrial function impairments (Minnock et al., 2022) or in T2D patients with early-onset T2D following an exercise intervention which diverges from current guidelines for exercise prescription in diabetes (Burns et al., 2007; Kanaley et al., 2022).

While T1D and T2D differ in physiopathology, mechanisms of VO_2_peak impairment, medication, and clinical outcomes, current clinical recommendations for exercise prescription are similar in many aspects for both conditions (Kanaley et al., 2022; Riddell et al., 2017). The general recommendations for subjects with T1D and T2D involve a combination of aerobic and resistance exercise. Evidence indicates that most health-related benefits and risks reduction can be achieved through 75–150 min/week of vigorous-intensity or 150–300 min/week of moderate-intensity physical activity. Additionally, high-intensity interval training (HIIT) has been shown to be feasible and more time-efficient than moderate-intensity training, while also being superior in improving VO_2_peak, body composition, mitochondrial functioning, insulin sensitivity, and glycemic control in patients with T1D and T2D (Kanaley et al., 2022; Murillo et al., 2022). Also, the higher intensity of training reached during HIIT increase the activation of AMP-kinase, a signalling protein involved in mitochondrial turnover (MacInnis and Gibala, 2017), but also in glucose homeostasis by activating non-insulin dependent pathways for GLUT-4 translocation (Marcinko et al., 2015). Moreover, the HIIT-induced circulating lactate levels and catecholamines inhibit the insulin-mediated glucose uptake and stimulate hepatic gluconeogenesis, thereby mitigating glycemic depletion during exercise (Guelfi et al., 2005). This limits the risk of exercise-related hypoglycaemia, a common concern in patients with T1D, and some with T2D, which can lead to exercise avoidance (Riddell et al., 2017).

As such, the aim of the present study is to evaluate if patients with well-controlled, uncomplicated T1D and T2D respond similarly to concurrent HIIT and RT, compared to age- and gender-matched healthy adults. The present investigation will focus on the main health-related fitness parameters such as aerobic capacity, body composition, lipid profile and glycemic control.

## 2 Methods

### 2.1 Participants

The present case-control study included sedentary adults with T1D and T2D, under stable medication and glycemic control over the last 3 months, and sex- and age-matched healthy nondiabetic controls (respectively HC1 and HC2). A preliminary anamnesis was taken for medical history and medication. In addition, patients’ endocrinologists confirmed the medical history and medication based on medical records and certified that the patients met the necessary criteria for participation in the study. Exclusion criteria were: contraindication to physical exercise, cardiac, pulmonary or vascular disease, severe diabetes complications (e.g., macroalbuminuric nephropathy, proliferative retinopathy, diabetic neuropathy), or >2 h/week of structured physical activity at moderate to high intensity. The global physical activity questionnaire (GPAQ) as completed to assess the last exclusion criterium.

All participants received written information regarding the protocol and had the opportunity to ask questions before giving written consent. The experimental protocol was approved by the Erasmus hospital ethical committee, Brussels, Belgium (reference P2018/387).

**FIGURE 1:**
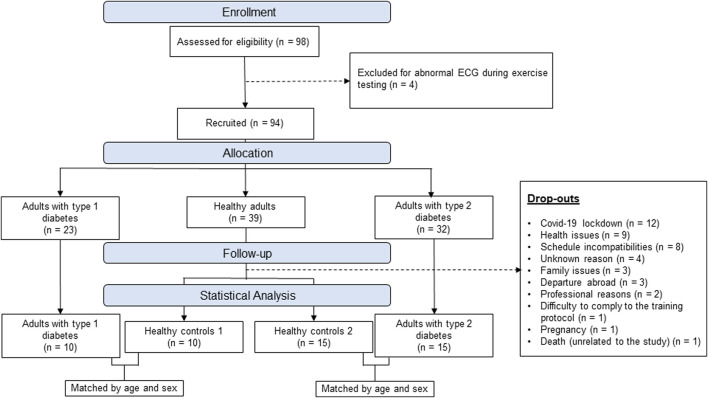
Study flow diagram

As presented in the flowchart Figure 1, 98 participants were initially enrolled (23 T1D, 32 T2D, 43 healthy controls) however 4 of them were excluded for abnormal electrocardiogram (ECG) and 44 dropped-out during the exercise intervention for various reasons. After data collection, matching of subjects was done manually, by an independent investigator, through a table that only included participants’ code, sex and age. Matching was then done blinded by matching healthy controls and patients with diabetes based on sex and age + - 5 years. The baseline characteristics of the 4 groups are presented in Table 1.

**TABLE 1:** Participants baseline characteristics.

	T1D (n = 10)	HC1 (n = 10)	T2D (n = 15)	HC2 (n = 15)
Age (years)	43 ± 13	43 ± 13	53 ± 8	54 ± 11
Sex (Female/Male)	7/3	7/3	4/11	4/11
BMI (kg/m^2^)	27 ± 5	24 ± 3	31 ± 5*	27 ± 5
Duration of diabetes (years)	13 ± 5	-	10 ± 5	-
HRmax	165 ± 13	176 ± 17	164 ± 15	163 ± 11
HbA1c (%)	8.1 ± 2.3*	5.2 ± 0.3	7.0 ± 1.0*	5.4 ± 0.3
Physical activity habits				
Intense PA (Met.min)	0 [0; 0]	0 [0; 0]	0 [0; 0]	0 [0; 0]
Moderate PA (Met.min)	440 [210; 1,050]	1,170 [660; 1760]	1,680 [240; 3,840]	1,140 [500; 2,280]
Comorbidities				
Hypertension (controlled)	0	0	6	4
Asthma	0	0	1	1
Hyperlipidemia	0	0	6	4
Diabetes pharmacological treatment				
Insulin	10	-	4	-
Metformin	1	-	14	-
SGLT-2 inhibitors	0	-	3	-
GLP-1 analog	0	-	1	-
Sulfonylureas	0	-	2	-
Glinides	0	-	1	-

Data presented as Mean ± Standard Deviation or Median [Q1; Q3],*p < 0.05. Abbreviations: HbA1c, Glycated hemoglobin; HC 1, Healthy Control group matched to patients with T1D; HC 2, Healthy Control group matched to patients with T2D; HRmax, Maximal Heart Rate; GLP-1, Glucagon-like peptide 1; MET, Metabolic equivalent Task. PA, physical activity; SGLT-2, Sodium-Glucose Co-Transporter Type 2.

### 2.2 Study design

**FIGURE 2:**
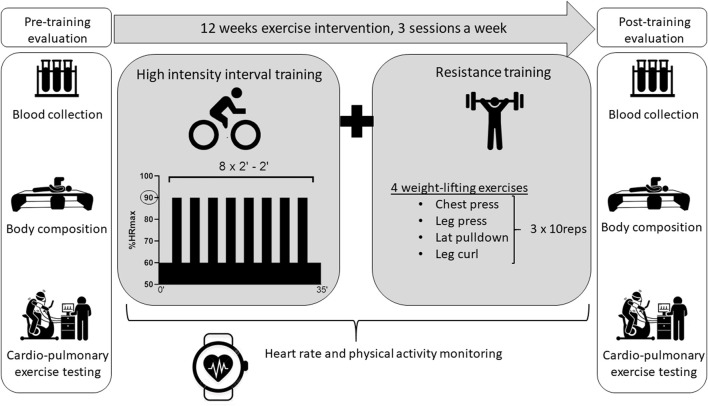
Protocol design.

All participants were referred to the laboratory on 2 occasions: prior and after a 12-week exercise intervention combining HIIT and RT. For minimal diurnal variation, tests were performed at the same time of the day in the morning. Blood sample and body composition assessment were performed first after an overnight fast. They were followed by a 30-min rest period to eat. Participants subsequently performed a cardio-pulmonary exercise test (CPET) on a cyclo-ergometer. The study protocol is illustrated in Figure 2. To avoid confounding factors and isolate training impacts, participants were asked to maintain their dietary habits and daily life activities during the entire intervention.

### 2.3 Measurements

#### 2.3.1 Blood analyses

Blood samples were analyzed by the laboratory of the Erasmus hospital, Brussels to assess blood lipids (high-density lipoprotein cholesterol [HDL-C], low-density lipoprotein cholesterol [LDL-C], total cholesterol and triglyceride levels) and glycemic profile (fasting glucose level, glycated hemoglobin [HbA1c], and c-peptide levels). Homeostasis model assessment of insulin resistance (HOMA-IR) was calculated in T2D patients and HC2.

#### 2.3.2 Body composition

After height and weight measurements, the participants’ body composition was assessed using dual energy X-ray absorptiometry (DXA; Lunar Prodigy, GE Healthcare, Madison, WI, United States) and analyzed using the enCORE software (version 15.0). The additional COREscan software was used for estimation of visceral adipose tissue (VAT).

#### 2.3.3 Aerobic exercise capacity

Aerobic capacity was assessed during an incremental CPET performed on an electrically braked cyclo-ergometer (Ergoselect 100; Ergoline, Bitz, Germany) in accordance with international recommendations (Wasserman et al., 1973). Oxygen uptake (VO_2_), carbon dioxide production (VCO_2_) and ventilation (VE) were collected breath by breath through a facial mask. Expiratory gas was analyzed using a metabolic system (Ergocard^®^; Medisoft, Dinant, Belgium) calibrated with room air and standardized gas. The testing protocol started with a 3-min warm up at 20 W for women and 30 W for men followed by 15 W/min increment for women and 20 W/min increment for men until exhaustion. A 12-lead ECG and finger pulse oximeter monitored continuously heart rate (HR) and peripheral arterial oxygen saturation respectively.

Since a plateau of VO_2_ was not observed in all participants, VO_2_peak was chosen as an indicator of maximal aerobic capacity. VO_2_peak was considered as achieved when two of the following criteria were met: (1) <100 mL/min VO_2_ increase with a further increase in workload, (2) a respiratory exchange ratio (RER) greater than 1.15, (3) inability to maintain pedaling frequency >50 rpm and (4) achievement of age-predicted maximal heart rate (HRmax). HR_max_ was considered as the highest value of HR achieved during CPET. VO_2_peak was defined as the highest VO_2_ value, averaged over 8 breath cycles, reached during the final stage of the incremental protocol. Maximal O_2_pulse was calculated as VO_2_/HR at VO_2_peak and was used as a surrogate for maximal stroke volume. The first ventilatory threshold (VT1) was determined by the V-slope and the ventilatory equivalent methods. The VE/VCO_2_ slope (i.e., ventilation required to exhale 1 L/min of CO_2_) was used to assess ventilatory efficiency. The HR/VO_2_ slope was calculated to assess the chronotropic response to exercise and the VO_2_/Wslope to assess the VO_2_ kinetics (Wasserman et al., 1973).

### 2.4 Exercise intervention

All participants performed an identical training protocol illustrated in Figure 2. Each training session was conducted 3 times a week, on nonconsecutive days, for 12 weeks in a fitness center nearby the participant’s home or workplace. The training program combined both aerobic (HIIT) and strength training components. The HIIT protocol consisted in a 3-min warm-up on a stationary bike, followed by 8 bouts of 2 min at 90% of HR_max_ measured during CPET, interspaced by 2 min of active recovery at a HR corresponding to VT1. The strength training component included 4 resistance exercises focusing on main muscle groups (i.e., chest press, leg press, lateral pulldown and leg curl). For each resistance exercise, 3 sets of 10 repetitions were performed at a load corresponding to 10 repetitions maximum, which was determined during the first session.

The first training sessions were supervised by an investigator who explained the protocol, calibrated the workload for each exercise, and ensured the participants fully understood the program. The importance of adherence to the protocol and the importance of physical activity tracking were also explained. Participants with diabetes also received information regarding the prevention of exercise induced hypoglycemia and eventual insulin bolus adjustment, as recommended in guidelines (Kanaley et al., 2022; Riddell and Peters, 2023). The supervision continued until participants became fully autonomous with weekly phone contact ensuring participant compliance and follow-up. Additionally, a supervised session was scheduled every 2 weeks to monitor load progression and ensure proper execution of the exercises. The remaining training sessions were carried out autonomously while HR and energy expenditure were recorded through a physical activity tracker (Polar M430 or Polar Ignite, Polar, Finland). This remote supervision via physical activity trackers has previously been shown to be realistic with a positive impact on adherence and compliance in patients with T1D (Scott et al., 2020). Moreover, it allowed us to verify both the completion of the sessions and their frequency through the application’s integrated training schedule. Data were exported to the Polar Flow app for assessment of total daily energy expenditure, training related energy expenditure, peak and mean HR during sessions expressed in % of HR_max_. Time spent in different intensity zones defined by Polar (light: 60–70%HR_max_, moderate: 70%–80% HR_max_, high: 80%–90% HR_max_, or maximal: >90% HR_max_) have been extracted. Due to technical issues, data of 3 healthy controls and 4 participants with T2D could not be retrieved.

### 2.5 Statistical analysis

Statistical analyses were carried out for comparisons between T1D and HC1 and between T2D and HC2. Distribution of the data was first assessed by a Shapiro-Wilk test. Inter-group baseline characteristics, total daily energy expenditure, training-related energy expenditure, and the highest and mean HR during training sessions were analyzed using a student t-test or a Mann-Whitney U test depending on the distribution.

A linear mixed model analysis was used to assess the intra-group effects of exercise training, and to compare inter-group effects between patients with T1D or T2D and their matched healthy controls. A prior Levene test was performed to assess homogeneity of variance.

According to data distribution, Spearman or Pearson’s correlation coefficient were used to analyse the association between variables.

Statistics were conducted using R (version 3.6.3) and *p* values below 0.05 were considered as significant. Graphics were created using GraphPad Prism (version 8).

An *a priori* power calculation was made and gave a sample size of 15 participants in each group. Since this sample size could not be achieved in the T1D group, an analysis of the study power, based on VO_2_peak differences before and after treatment, was performed using the open resource GLIMMPSE 3.0 (Kreidler et al., 2013). VO_2_peak was chosen as the main outcome since it was previously reported as blunted in patients with diabetes. For T1D the power was between 85% and 90% with a sample of 10 participants and, for T2D, the power was greater than 95% with 15 participants.

## 3 Results

Apart from two participants who encountered minor osteoarticular pain, leading to their withdrawal from the study, no other adverse events related to the training were observed.

### 3.1 HR and energy expenditure recordings

Energy expenditure (daily and related to training) and chronotropic responses during exercise training sessions (mean and peak HR and time spent in different intensity zones) are presented in Table 2 with no differences between T1D and HC1 or T2D and HC2.

**TABLE 2:** Physical activity tracker recordings.

	T1D (n = 10)	HC1 (n = 9)	*p*	T2D (n = 11)	HC2 (n = 13)	*p*
Energy expenditure (EE)						
Total daily EE (kcal)	2,545 ± 464	2,684 ± 517	0.56	2,744 ± 911	2,783 ± 552	0.65
Training related EE (kcal/session)	405 ± 63	412 ± 136	0.88	671 ± 458	447 ± 126	0.21
Heart rate (HR) during training sessions						
Highest HR (%HRmax)	92 ± 4	89 ± 4	0.13	90 ± 6	92 ± 4	0.36
Mean HR (%HRmax)	74 ± 7	71 ± 4	0.27	70 ± 5	73 ± 5	0.27
Training zone time						
Maximal intensity (min)	1.2 ± 2.5	1.1 ± 1.8	0.92	5.8 ± 6.2	3.3 ± 2.9	0.47
High intensity (min)	9.3 ± 6.5	9.4 ± 6.1	0.98	14.7 ± 6.2	13.6 ± 5.5	0.64
Moderate intensity (min)	17.7 ± 5.3	19.3 ± 3.5	0.44	18.1 ± 4.9	20.7 ± 16.1	0.69
Light intensity (min)	15.4 ± 5.0	18.1 ± 5.4	0.27	10.7 ± 5.6	12.6 ± 6.4	0.46

EE, Energy expenditure; HC 1, Healthy Control group matched to patients with T1D; HC, 2. Healthy Control group matched to patients with T2D; HR, Heart rate; T1D, Patients with type 1 diabetes; T2D, Patients with type 2 diabetes.

### 3.2 Glycemic and lipid profiles

Blood metabolic features are presented in Table 3. At baseline, patients with T1D exhibited higher fasting glucose, HbA1c and lower C-peptide as compared to HC1 (all *p* < 0.001). Patients with T2D displayed higher fasting glucose, HbA1c, triglycerides and HOMA-IR as compared to HC2 (all *p* < 0.05). All other blood parameters were similar between patients and healthy controls. Glycemic and lipid profiles remained unaffected by the exercise intervention. HOMA-IR improved with training in patients with T2D (*p* < 0.05). The decrease in triglycerides in T2D was negatively correlated to the baseline level (r = −0.5, *p* = 0.004).

**TABLE 3:** Blood metabolic features.

		Pre	Post		Pre	Post	Interaction P
Cholesterol (mg/dL)	T1D	194 ± 40	208 ± 42	HC 1	191 ± 26	196 ± 36	0.42
	T2D	178 ± 46	172 ± 36	HC 2	199 ± 37	197 ± 33	0.78
Triglycerides (mg/dL)	T1D	110 ± 73	103 ± 42	HC 1	91 ± 32	99 ± 47	0.22
	T2D	184 ± 104^†^	158 ± 68	HC 2	120 ± 49	110 ± 35	0.49
HDL-C (mg/dL)	T1D	75 ± 27	78 ± 22	HC 1	62 ± 12	64 ± 11	0.68
	T2D	43 ± 11^†^	45 ± 12	HC 2	58 ± 15	59 ± 14	0.71
LDL-C (mg/dL)	T1D	97 ± 22	112 ± 29	HC 1	111 ± 27	114 ± 36	0.19
	T2D	98 ± 41	95 ± 30	HC 2	117 ± 36	116 ± 32	0.93
Fasting glucose (mg/dL)	T1D	198 ± 98^†^	201 ± 100	HC 1	95 ± 8	89 ± 11	0.75
	T2D	160 ± 41^†^	153 ± 54	HC 2	97 ± 9	95 ± 11	0.52
HbA1c (%)	T1D	8.4 ± 2.4^†^	8.3 ± 2.2	HC 1	5.2 ± 0.3	5.2 ± 0.3	0.50
	T2D	7.0 ± 1.0^†^	6.8 ± 0.9	HC 2	5.5 ± 0.3	5.4 ± 0.3	0.41
C-peptide (nmol/L)	T1D	0.08 ± 0.05^†^	0.10 ± 0.09	HC 1	0.67 ± 0.20	0.66 ± 0.29	0.36
	T2D	1.02 ± 0.36	0.99 ± 0.67	HC 2	0.89 ± 0.44	0.85 ± 0.38	0.78
HOMA-IR	T1D	-	-	HC 1	-	-	-
	T2D	3.48 ± 1.66^†^	3.06 ± 1.78*	HC 2	2.26 ± 1.97	2.10 ± 1.57	0.83

^†^ *p* < 0.05 intergroup difference at baseline. *p < 0.05 intra-group difference after training. HbA1c. Glycated hemoglobin; HC 1, Healthy Control group matched to patients with T1D; HC 2, Control group matched to patients with T2D; HDL-C, High density lipoprotein cholesterol; HOMA-IR, Homeostatic model assessment of insulin resistance; LDL-C, Low density lipoprotein cholesterol; T1D, Patients with type 1 diabetes; T2D, Patients with type 2 diabetes.

### 3.3 Body composition

**FIGURE 3:**
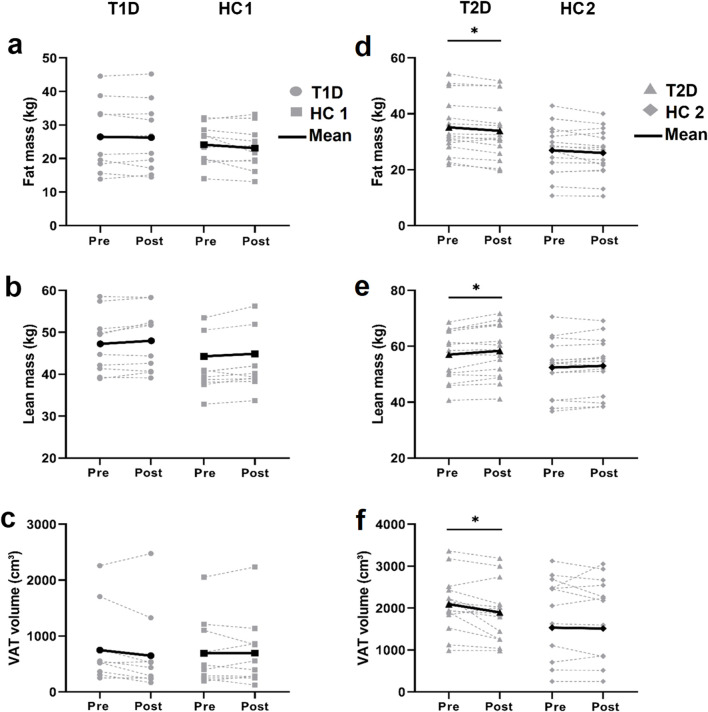
DEXA Body composition assessment Individual (grey lines) and mean (black lines) values of total fat mass **(A, D)**. total lean mass **(B, E)** and visceral adipose tissue (VAT) volume **(C, F)** before and after training in patients with type 1 diabetes (T1D) and their healthy controls (HC1; left panels) and in patients with type 2 diabetes (T2D) and their healthy controls (HC2; right panels). *p < 0.05; different from baseline value within the group.

At baseline, T1D and HC1 participants exhibited similar total fat mass, lean mass and VAT, which remained unchanged after training (Figure 3A–C). In contrast, patients with T2D had a higher fat mass at baseline (Figure 3D). Following exercise training, fat mass and VAT decreased, and lean mass increased (Figure 3D–F) in patients with T2D. However, the inter-group effect was not statistically significant (*p* > 0.05).

### 3.4 Aerobic exercise capacity

**FIGURE 4:**
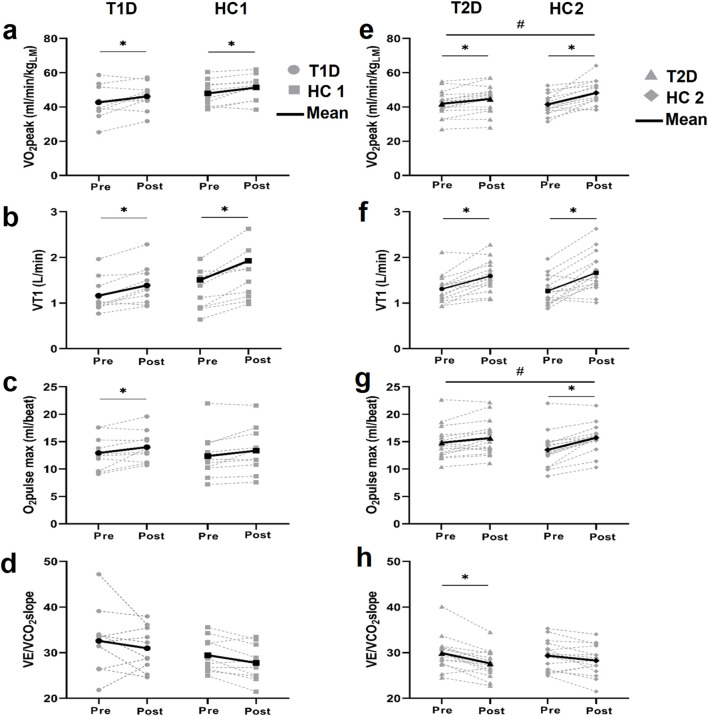
Cardio-respiratory fitness assessment Individual and mean values of VO_2_peak **(A, E)**. the first ventilatory threshold (VT1) **(B, F)**. the maximal O_2_pulse **(C, G)** and the VE/VCO_2_slope **(D, H)** before and after training in patients with type 1 diabetes (T1D) and their healthy controls (HC1) (left panels) and in patients with type 2 diabetes (T2D) and their healthy controls (HC2) (right panels). *p < 0.05 different from baseline value within the group, #*p* < 0.05 difference in the training response between groups.

CPET results are presented in Figure 4 and Table 4. At baseline, patients with diabetes and controls had similar aerobic capacity (all *p* > 0.05).

**TABLE 4:** Cardiorespiratory fitness assessment.

	Group	Pre	Post	Group	Pre	Post	Interaction P
Wmax (Watts)	T1D	159 ± 56	178 ± 54.2*	HC1	183 ± 58	198 ± 57*	0.49
	T2D	179 ± 45	208 ± 52*	HC2	186 ± 48	212 ± 50*	0.43
VO_2_peak (L/min)	T1D	2.06 ± 0.71	2.23 ± 0.60*	HC1	2.14 ± 0.67	2.32 ± 0.68*	0.75
	T2D	2.37 ± 0.51	2.58 ± 0.53*	HC2	2.18 ± 0.56	2.54 ± 0.51*	0.10
VO_2_peak/BW (mL/kg/min)	T1D	27.2 ± 8.3	29.7 ± 8.1*	HC1	30.1 ± 6.8	32.7 ± 7.1*	0.81
	T2D	25.4 ± 6.8	27.7 ± 7.2*	HC2	26.8 ± 5.1	31.6 ± 5.7*	**0.03**
VO_2_peak/LM (ml/kg_LM_/min)	T1D	42.7 ± 9.9	46.2 ± 7.8*	HC1	48.0 ± 7.6	51.3 ± 7.3	0.67
	T2D	41.9 ± 7.7	44.7 ± 8.2*	HC2	41.5 ± 5.9	48.2 ± 7.1*	**0.03**
HRmax (bpm)	T1D	166 ± 13	165 ± 13	HC1	177 ± 17	175 ± 14	0.97
	T2D	164 ± 15	165 ± 16	HC2	163 ± 11	161 ± 11	0.45
VEmax (L/min)	T1D	91 ± 30	98 ± 32*	HC1	95 ± 27	99 ± 25	0,45
	T2D	100 ± 19	104 ± 21	HC2	95 ± 24	103 ± 22*	0.48
Maximal O_2_ pulse (ml/beat)	T1D	12.9 ± 3.2	14.0 ± 2.9*	HC1	12.4 ± 4.2	13.4 ± 4.3	0.66
	T2D	14.8 ± 3.1	15.7 ± 3.2	HC2	13.5 ± 3.3	15.7 ± 2.7*	**0.048**
Maximal RER	T1D	1.22 ± 0.06^†^	1.23 ± 0.07	HC1	1.29 ± 0.07	1.26 ± 0.07	0.07
	T2D	1.22 ± 0.11	1.24 ± 0.08	HC2	1.28 ± 0.08	1.26 ± 0.04	0.15
VT1 (L/min)	T1D	1.17 ± 0.37	1.39 ± 0.42*	HC1	1.26 ± 0.43	1.61 ± 0.53*	0.22
	T2D	1.31 ± 0.30	1.59 ± 0.34*	HC2	1.27 ± 0.32	1.67 ± 0.44*	0.38
VT1(%VO_2_max)	T1D	59 ± 11	62 ± 10	HC1	59 ± 10	69 ± 5*	0.18
	T2D	56 ± 7	62 ± 10*	HC2	59 ± 8	66 ± 11*	0.97
VE/VCO_2_ slope	T1D	33 ± 7	31 ± 5	HC1	30 ± 4	29 ± 5	0.68
	T2D	30 ± 4	28 ± 3*	HC2	29 ± 3	29 ± 4	0.07
VO_2_/W slope (mL/kg/min/W)	T1D	9.9 ± 1.1	9.9 ± 1.4	HC1	9.9 ± 1.0	8.9 ± 2.1	0.30
	T2D	10.6 ± 2.1	10.2 ± 1.9	HC2	9.5 ± 1.2	9.8 ± 1.2	0.22
HR/VO_2_ slope (beats/mL/kg)	T1D	3.3 ± 0.8	2.9 ± 0.6	HC1	4.0 ± 1.0	3.4 ± 0.7	0.97
	T2D	3.7 ± 0.6	3.7 ± 0.8	HC2	3.7 ± 1.0	3.2 ± 0.6*	0.11

^†^ *p* < 0.05 intergroup difference at baseline. *p < 0.05 intra-group difference after training. BW, Body weight; HC 1, Healthy Control group matched to patients with T1D; HC, 2; HR, Heart rate; Control group matched to patients with T2D; LM, Lean Mass; RER, Respiratory exchange ratio; T1D, Patients with type 1 diabetes; T2D, Patients with type 2 diabetes; VE, Ventilation; VCO_2_, Carbon dioxide production; VO_2_peak, Peak oxygen output; VT1, First ventilatory threshold; Wmax. maximal power output. Bold numbers indicate significant interactions.

After training, patients with T1D and HC1 similarly improved VO_2_peak (T1D: +11%, HC1: +9%), VT1 (T1D: +20%, HC1: +30%) and maximal O_2_pulse (T1D: +10%, HC1: +9%) (interaction *p* > 0.05), while VE/VCO_2_, HR/VO_2_ (not shown) and VO_2_/W (not shown) slopes remained unchanged.

After training, patients with T2D and HC2 showed similarly improvement of VT1 (T2D: +23%, HC2: +34%, both *p* < 0.05, interaction *p* = 0.38), and VE/VCO_2_slope (T2D: 7% (*p* < 0.05); HC2: 4% (*p* > 0.05), interaction *p* = 0.9). Interestingly, patients with T2D exhibited less improvement in VO_2_peak (T2D:+9% (*p* < 0.05), HC2: +18% (*p* < 0.05), interaction *p* < 0.05) and O_2_pulse at maximal exercise (T2D: +6% (*p* > 0.05), HC2: +18% (*p* > 0.05), interaction *p* < 0.05).

## 4 Discussion

To our best knowledge, this study is the first to investigate simultaneously the effects of an identical concurrent HIIT and resistive exercise intervention, performed autonomously, on lipid profile, glycemic control, body composition and aerobic capacity in patients with T1D and T2D compared to matched healthy nondiabetic adults.

The concurrent HIIT and RT yielded comparable improvements in VO_2_peak, maximal O_2_pulse and VT1 for patients with T1D and healthy controls, with no influence on body composition or lipid and glycemic profiles. Patients with T2D also increased cardio-respiratory fitness, evidenced by improvements in VO_2_peak, VT1 and VE/VCO_2_ after training, but VO_2_peak increased to a lesser extent compared to matched healthy controls. Nevertheless, patients with T2D had additional health benefits from the exercise training by reducing their cardio-vascular risk and metabolic profile (improvement of body composition and insulin sensitivity). Those benefits are of considerable clinical value in the management of T2D and for the prevention or postponement of long-term complications.

### 4.1 Training response in patients with T1D

In the present study, otherwise healthy patients with T1D exhibited body composition similar to that of their matched healthy controls, and their baseline lipid profile was within normal values. In both groups, body composition and blood parameters were not modified by the exercise intervention. Although a recent review/meta-analysis highlighted a training-induced reduction in body weight, LDL and triglycerides in patients with T1D, no changes were seen in the present normal weight and eulipidemic participants (Ostman et al., 2018). The lack of change in LDL and triglycerides in our participants could be explained by the normal values at baseline, making significant changes less likely in response to exercise training. The absence of significant changes in body composition is in agreement with previous studies reporting that exercise often fails to reduce total body fat mass (Swift et al., 2018) and VAT (Maillard et al., 2016) over a limited period of time especially in participants with limited excess and when exercise training was not combined with dietary intervention.

Previous reports described a VO_2_peak alteration in patients with T1D and poor glycemic control (Turinese et al., 2017; Moser et al., 2018) which was attributed to impaired insulin sensitivity, micro-vascular defects, poor vascular reactivity, and skeletal muscle mitochondrial dysfunction (Riddell and Peters, 2023). Also, poor glycemic control, leads to an increase in HbA1c and advanced glycation end products (AGEs) levels (Shen et al., 2020). AGEs alters physiological properties of proteins and induces vascular changes, both leading to diastolic dysfunction, thereby reducing VO2 peak (Willemsen et al., 2011). In addition, patients with T1D compensate for impaired carbohydrate metabolism by increasing lipid catabolism during exercise, resulting in a lower RER (Turinese et al., 2017). Furthermore, chronic hyperglycemia leads over time to an impaired chronotropic response related to a desensitization of cardiac ß-adrenergic receptors on over-secretion of catecholamines (Moser et al., 2018; Eckstein et al., 2021). Exercise chronotropic response in patients with T1D has been found to be altered and correlated to HbA1c if above 7.9% (Moser et al., 2018). In the present study, patients with T1D exhibited identical physical activity levels and unaltered VO_2_peak, RER, HR/VO_2_ slope and HRmax at baseline compared to HC1. Only 30% of our participants with T1D had HbA1c levels above 7.9%, supporting the idea that improving glycemic control may minimize impairment of cardio-metabolic health, cardio-vascular response, and aerobic performance (Riddell and Peters, 2023). The inability to detect clinical or subclinical impairments in cardiovascular or muscular responses to exercise between patients with T1D and controls may also be related to the heterogeneity within the T1D group at baseline as indicated by a considerable variability in aerobic capacity (VO_2_peak), muscle oxidative capacity (VT1), maximal stroke volume (O_2_ pulse max), chronotropic response (HR/VO_2_ slope), and ventilatory response (VE/VCO_2_ slope) (Figure 4).

Training-induced VO_2_peak improvements in patients with T1D and HC1 are consistent with previous studies reporting VO_2_peak improvement after HIIT in patients with T1D (Scott et al., 2019) and healthy adults (MacInnis and Gibala, 2017). In the present study, improvement of VO_2_peak was similar in both groups, in consistency with previous observations. However, a recent study found a lower VO_2_peak increase to a 12-week concurrent training in patients with T1D compared to healthy individuals, despite similar baseline values (Minnock et al., 2022). Authors attributed the altered response to the existence of a pre-training, subclinical myopathy (i.e., mitochondrial dysfunction, inflammation, and muscle atrophy) which was not restored through training (Minnock et al., 2022). In our study, no patient was complaining or diagnosed with muscular deconditioning. Also, the time since the onset of T1D was shorter in our study (13 ± 5 years versus 18 ± 9 years). Indeed, T1D duration has been associated with muscle mitochondrial dysfunction (Heyman et al., 2020). The extent to which T1D mitochondrial alteration affects VO_2_peak adaptation to training remains to be clarified. Nevertheless, those observations globally highlight the importance of glycemic control over time to preserve aerobic capacity and its response to training.

### 4.2 Training response in patients with T2D

Physical activity is an important component in the management of T2D. Interventions combining HIIT and RT have been shown to induce superior benefits than endurance or RT alone in inducing weight-loss and improving glycemic control, insulin sensitivity and cardio-vascular risk among patients with T2D with overweight/obesity (Kanaley et al., 2022). Combined training is also known to improve LDL-C, HDL-C, total cholesterol and triglyceride levels in a context of dyslipidemia or T2D (Amanat et al., 2020; Zhao et al., 2021). At baseline, the patients with T2D had higher triglyceride and lower HDL-C levels with no significant change after training. In line with previous studies, training-induced triglyceride changes were correlated to baseline levels, with participants with higher baseline triglycerides experiencing the largest decrease (Amanat et al., 2020; Mann et al., 2014). HbA1c levels were not affected by training, but HOMA-IR decreased in patients with T2D. A recent meta-analysis demonstrated the benefits of combined exercise in overweight/obese T2D patients on dyslipidemia, insulin sensitivity and glycemic control with a great variability in the HbA1c response (Zhao et al., 2021). According to Kanaley et al., a weight loss of >5% is required to achieve beneficial effects on HbA1c and blood lipids in most individuals (Kanaley et al., 2022). Taking into consideration that the current study demonstrate a change in body weight ranging from +3% to −3% in patients with T2D after training, this potentially explains the absence of a significant change in HbA1c after training. This, however, does not take the body composition into account. Fat loss, and more specifically VAT loss, remains one of the main objectives in T2D (Magkos et al., 2020). Hence, a moderate decrease of total and VAT was observed in T2D patients, which is important to alleviate metabolic dysfunction, insulin resistance and cardio-vascular risk. Although the observed changes were small, the occurrence after a short training period, and without dietary intervention, is encouraging. In addition, improvement of lean body mass is of importance for basal metabolic rate, long-term weight control (Pollock et al., 2000) and blood glucose homeostasis (Amanat et al., 2020).

Improvement in VO_2_peak after HIIT has been consistently reported in patients with T2D (Liu et al., 2019). However, in the present study, the mean magnitude of increase in VO_2_peak for patients with T2D was less pronounced compared to age/sex-matched healthy controls with similar baseline VO_2_peak levels. This result aligns with findings from Pandey et al. who reported, in a large study, that less than 37% of T2D patients undergoing structured exercise training achieved a ≥5% improvement in VO_2_peak (Pandey et al., 2015). While this provides valuable information, their study did not incorporate HIIT nor include a direct comparison with healthy individuals subjected to the same training regimen, as was done in the present study. The underlying reason for this altered response is still not fully understood. Lower daily or training-related energy expenditure cannot be incriminated as they were identical in both groups. Impaired training responses of VO_2_peak and maximal O_2_Pulse are suggestive of a cardio-vascular origin, especially if associated with unaltered responses of indices sensitive to peripheral muscle changes, such as VT1 and RER (Wasserman et al., 1973). Previous studies have evoked chronotropic incompetence, left ventricular hypertrophy, and adverse cardiac remodelling to explain the compromised training benefits observed in individuals with T2D (Pandey et al., 2015). It seems reasonable to assume that a combination of these factors, along with the accumulation of multiple cardiovascular risk factors and subclinical myocardial, vascular, or endothelial dysfunction, contribute to the attenuated response and may not be fully reversible through training alone (Nesti et al., 2020).

Other authors have proposed a peripheral muscle limitation associated with abnormalities in pathways regulating training-induced mitochondrial adaptations (Hernández-Alvarez et al., 2010). Notably, Burns and colleagues observed altered VO_2_peak trainability following 12 weeks of moderate intensity continuous training although the underlying mechanisms were minimally explored (Burns et al., 2007). Subsequently, Hernandez-Alvarez and colleagues reported that PGC1-α pathways were altered in T2D following 12 weeks of MICT, potentially explaining the blunted VO_2_peak response (Hernández-Alvarez et al., 2010). However, considering the impact of exercise intensity on training-induced adaptations, and particularly on PG1-α activity (MacInnis and Gibala, 2017), data obtained following MICT cannot be fully extrapolated to the present concurrent HIIT protocol.

Metformin, the most prescribed glucose-lowering medication and taken by 93% of our T2D study participants, has been shown to attenuate the training-enhanced peripheral insulin sensitivity and mitochondrial adaptations, and alters VO_2_peak improvements in adults with prediabetes (Konopka et al., 2019; Walton et al., 2019). Indeed, both acute metformin intake and physical activity reduces oxidative stress and improves AMPK activity (Martín-Rodríguez and de Pablos-Velasco, 2020). However, the mechanisms behind their interaction remain unclear and there is an ongoing debate in literature whether metformin either inhibits or improves mitochondrial function. It is plausible than when combined, metformin may interfere with the beneficial adaptations from physical activity, leading to diminished training-induced improvements (Martín-Rodríguez and de Pablos-Velasco, 2020). Therefore, in the present study, metformin might have contributed to the inter-individual variability in exercise-induced improvements in insulin sensitivity, glycemic response, and VO_2_peak (Malin and Stewart, 2020). More research is however needed to clarify those interactions.

Noteworthy, T2D is a highly heterogeneous disease, with certain clusters carrying a higher risk of specific complications (Ahlqvist et al., 2018). This variability may affect training-induced adaptations, leading to different responses based on the underlying pathophysiological conditions. Larger-scale exercise intervention studies that stratify participants by cluster could provide valuable insights into the determinants of impaired exercise responses in diabetes.

It should nonetheless be emphasized that the concurrent training was largely beneficial in patients with T2D. Although blunted, VO_2_peak still increased, and body composition, lipid profile, insulin resistance, VT1, and ventilatory efficiency improved. The latter is of major clinical interest since the VE/VCO_2_ slope represents the ventilatory efficiency or chemosensibility and is an independent risk factor for cardiovascular disease (Nayor et al., 2020). Moreover, VE/VCO_2_ slope was recently reported as being lower in patients in T2D remission as compared to patients with active T2D (Bilak et al., 2023). VE/VCO_2_ slope reduction is therefore suggestive of an improved health-related fitness in patients with T2D.

This study has some limitations. The patients with diabetes, recruited from our university hospital, did not have major limitations at baseline, were well controlled and without complications. Therefore, the absence of differences between patients and healthy controls at baseline could be attributed to the absence of medical or physical limitations in our patients. The results can therefore not be extrapolated to patients with more severe limitations or worse glycemic control. Another limitation is that despite asking patients to keep their dietary habits, this was not monitored. However, it is noteworthy thar recording its diet may alter food intake, and bias related to self-report could happen unintentionally or to reduce burden (Shim et al., 2014).

## 5 Conclusion

The present study shows that a 12-week exercise intervention, combining HIIT and RT, performed autonomously by patients with T1D and T2D, is safe and efficient for improving VO_2_peak and VT1. Training-induced effects in patients with T1D included significant enhancements in VO2peak, VT1, and maximal workload, similar to those seen in healthy controls. However, patients with T2D exhibited a lower training-induced improvement in VO_2_peak and maximal O2pulse compared to healthy controls through mechanisms that still require further elucidation. Nonetheless, concurrent training provides notable additional health benefits for patients with T2D such as improvements in insulin sensitivity, blood lipids, body composition (reduced VAT and increased lean mass), and VT1 and smoothened ventilatory response to exercise. These benefits have considerable clinical value in the management of T2D by mitigating risk factors and preventing or delaying long-term complications.

## 6 Perspectives

The present findings carry significant implications for diabetes management and exercise interventions. First, the preserved aerobic capacity seen in patients with uncomplicated diabetes and good glycemic control underscores the importance of sustained glycemic control to mitigate diabetes-induced aerobic capacity impairment. Second, the contrasting training responses between T1D and T2D suggest the need for tailored exercise approaches. Patients with T1D can achieve similar training improvements to healthy adults, indicating the feasibility of exercise as a mitigating factor. In contrast, reduced trainability in patients with T2D regarding VO_2_peak is of clinical importance and must be taken into consideration during exercise prescription and monitoring in patients with T2D. Personalized interventions could focus on training strategies to counteract a reduced VO_2_peak response by increasing the volume or emphasizing efforts stimulating cardiovascular and/or skeletal muscle mitochondrial function. These targeted approaches could even more effectively reduce the rate of progression of the disease.

Thirdly, further research will be needed to identify diabetes-associated risk factors and diabetes complications that contribute to impaired trainability in patients with T2D.

Finally, our findings stimulate inquiries into the mechanisms underlying these diverse training responses, including insulin resistance, inflammation, substrate utilization, etc. A deeper understanding of the influence of these factors on trainability could refine and personalize exercise prescriptions in each diabetes subtype.

## Data Availability

The raw data supporting the conclusions of this article will be made available by the authors on reasonable demand, without undue reservation.
